# Unusual infections and thrombotic events in Cushing’s syndrome

**DOI:** 10.1007/s40618-024-02454-8

**Published:** 2024-10-01

**Authors:** Mattia Barbot, Martina Lazzara, Pierluigi Mazzeo, Francesca Pecori Giraldi

**Affiliations:** 1 Endocrinology Unit, Department of Medicine-DIMED, University Hospital of Padova, Padova, Italy; 2 Department of Clinical Sciences & Community Health, University of Milan, Via Commenda 19, Milano, Italy

**Keywords:** Cushing’s syndrome, Infections, Opportunistic infections, Thrombosis, Thrombophilia

## Abstract

The diagnosis of Cushing’s syndrome requires a high degree of suspicion, especially in patients in whom typical features are overshadowed by other ailments. These include, among others, widespread opportunistic infections or sepsis and venous or arterial thromboembolism.

This Review will summarize available data on patients presenting with severe infections or thrombotic events and the best approach to diagnosis.

**Supplementary Information:**

The online version contains supplementary material available at 10.1007/s40618-024-02454-8.

## Introduction

Cushing’s syndrome is a severe -although thankfully rare- endocrine disease. Its multifaceted presentation translates into signs and symptoms which may be easily recognized by the trained eye, e.g. moon facies, truncal obesity and muscle atrophy, and more unusual manifestations, such as susceptibility to opportunistic pathogens and thrombotic events. Thus, diagnosis of Cushing’s syndrome often requires complex and expert work-up.

This Review will describe the current knowledge on Cushing’s syndrome presenting with severe infection or acute thrombotic events and the best diagnostic approach for these patients.

An extensive MEDLINE search on the topic was performed independently by two authors (F.P.G. and M.B.), publications retrieved and read by all authors. The following search words were included: “Cushing’s syndrome, thrombosis, thrombotic events, infection, mortality”. Search terms were linked to Medical Subject Headings (MeSH) where available. Keywords and free words were used simultaneously. Additional articles were identified through manual research and study of meta-analyses, review articles and cross references. Any discrepancy was resolved by discussion.

## Hypercortisolism presenting with unusual infections

### Pathophysiology

Glucocorticoids are known to affect the immune system at different levels [1, 2]. On the one side, leukocytosis with increased neutrophils and reduced lymphocyte counts are among the hallmarks of excess cortisol exposure [3–5]. Increased neutrophil counts in the blood stream are in part due to reduced adhesion and extravasation at infection sites, indicative of blunted acute immune response. Dampening of macrophage function, crucial to phagocytosis, and reduction of natural killer (NK) cells [6, 7] -a subset of lymphocytes involved in cellular cytotoxicity- further affect the first line immune response. Glucocorticoids also modulate proinflammatory and antiinflammatory cytokines resulting varied changes to cytokine microenvironment and immune cell function [1, 2].

The adaptative immune response relies primarily on lymphocytes, T-cells for cellular immunity and B-cells for antibody production. Patients with Cushing’s syndrome present lower percentages of CD4 + lymphocytes –the so-called T-helper cells- and increased CD8 + cytotoxic lymphocytes [7], thus decreased CD4/CD8 ratio, a marker of immune activation. In clinical setting, low CD4 + counts have been described in a patient with ectopic ACTH secretion and multiple opportunistic infections [8].

In addition to these changes directly pertaining to the systemic immune response, patients with Cushing’s syndrome may also present the low-grade inflammation common to individuals with obesity, diabetes and the metabolic syndrome [9].

Of note, ectopic Cushing’s syndrome and adrenal cancer present increased immunosuppression related to malignancy itself, possibly exacerbated by chemotherapy and poor nutritional status in advanced metastatic disease [10–12].

### Epidemiology

The chronic corticosteroid excess leads to immunocompromised status with reduction of defences toward opportunistic infections [2]. The prevalence of severe infections ranges from 20% in Cushing’s disease to 50% in ectopic ACTH secretion [13, 14] (Table 1); overall, patients with Cushing’s syndrome present a 5-fold higher risk for infection [15]. Ultimately, infections and sepsis represent the second cause of death among patients with Cushing’s syndrome [15–23]. Indeed, Harvey Cushing himself reported the increased susceptibility to infection with high fatalities in his first series [24].

**Table 1:** Prevalence of severe infections in patients with active Cushing’s syndrome

Study	Number of patients	Indicator
Papakokkinou 2020 [21]	830 person – year for 3 years prior to diagnosis (all CD)	sepsis: none
Dekkers 2013 [12]	343 patients (211 CD, 132 adrenal)	infections: HR^a^ 2.6 (C.I. 1.1–6.4)
Broder 2015 [22]	1852 patients (all CD)	391 infections (21%)
Sarlis 2000 [16]	54 patients (all ectopic)	25 infections (46%)
Ilias 2005 [23]	90 patients (all ectopic)	46 infections (51%)
Ejaz 2011 [24]	43 patients (all ectopic)	10 infections (23%)
Toivanen 2021 [11]	60 patients (all ectopic)	33 infections (55%)
Mondin 2022 [25]	126 patients (all CD)	22 infections (17.5%)

CD: Cushing’s disease; ^a^HR: age-sex adjusted hazard ratio compared to general Danish population cohort; C.I. 95% confidence intervals

As mentioned above, patients with ectopic ACTH syndrome are more frequently affected by severe infections, often fatal [20] and this appears related to the degree of cortisol excess, usually more pronounced in ectopic disease [25]. In fact, the risk of infection is strongly correlated with the severity of hypercortisolism [20, 26, 27] across etiologies of Cushing’s syndrome and milder opportunistic infections, e.g. mucocutaneous fungal diseases, are probablyfrequent events. Indeed, a recent self-assessment survey revealed 3-fold higher prevalence of urinary tract infections and mycoses among patients with pituitary or benign adrenal Cushing’s syndrome [28]. Conversely, a pediatric study reported cutaneous fungal infections in only 20% of children [27] thus, the true prevalence of mild infections remains to be established. Of note, infections may be overlooked in patients with Cushing’s syndrome given that the classical acute response with fever and leukocytosis is blunted by cortisol excess itself [26].

### Clinical course

Severe infections usually monopolize the clinical presentation and dictate management. The main species of opportunistic pathogens reported in patients with Cushing’s syndrome are *Aspergillus fumigatus*, *Cryptococcus neoformans*, *Pneumocystis jirovecii*, *Nocardia asteroides* [2, 26, 29–48] (Supplementary Table 1). As with immunodeficient patients, these infections appear resistant to treatment, superimposed to other infections, widespread or in uncommon sites, and often fatal [8, 49]. Other, more common, agents -such as *mybacterium tuberculosis*, cytomegalovirus and Herpes Zooster virus- may present an uncommonly severe course in patients with Cushing’s syndrome. In fact, multiple mycobacterial abscesses [50] and disseminated Herpes Zooster [51] have been reported in patients with hypercortisolism.

Treatment of hypercortisolism, either medical and surgical, may be attempted to ameliorate the response to antibiotics and/or support therapy and, indeed, in several cases, led to resolution of the infection. Control of hypercortisolism is related to better outcomes and more rapid recovery from infections [26, 52].

Particular consideration should however be extended to patients with Cushing’s syndrome and *pneumocystis jirovecii* (formerly *pneumocystis carinii*) pneumonia. In most patients with Cushing’s syndrome reported so far [53–56] pneumonia developed after initiation of cortisol-lowering therapy. This timeline is in keeping with the so-called immune reconstitution inflammatory syndrome, i.e., an exaggerated inflammatory response to the fungus after reversal of immunosuppression.

### Who should be screened

The diagnosis of Cushing’s syndrome in patients presenting with infection requires a high degree of clinical suspicion by physicians unused to dealing with endocrine disorders. In fact, patients may attend the clinic for lung disease, skin lesions, gastrointestinal perforation, central nervous system mass, among others. Only careful history taking and physical examination will lead to suspect Cushing’s syndrome and avoid the pointless pursuit of other causes.

Patients presenting with opportunistic infections without a known underlying cause for immunosuppression should be evaluated for signs and symptoms of cortisol excess. Both severe systemic infections as well as chronic or recurring mild cutaneous infections should be considered suspicious for Cushing’s syndrome.

As mentioned above, the severity of infection is correlated with the degree of cortisol excess, thus patients with paraneoplastic hypercortisolism -due either to ectopic ACTH secretion or malignant adrenal tumor- are most likely to be affected. In these patients, rapid onset of the disease and preponderance of catabolic effects may mask typical signs of Cushing’s syndrome [2], such as obesity, facial plethora, and other features, e.g., hypokalemia, uncontrolled diabetes, may prompt the investigation [8, 43, 49]. Differential diagnosis encompasses some of the usual causes of non-neoplastic Cushing’s syndrome, in particular long-standing alcoholism, as well as causes of immunodeficiency, i.e., HIV infection. The latter is of particular interest given the abnormal fat accumulation which has been reported to occur in patients treated with antiretroviral drugs, i.e., “HIV lipodistrophy” [57].

### Diagnostic work-up

First line tests, i.e., urinary free cortisol, overnight suppression test, late-night salivary cortisol, should be used to screen for Cushing’s syndrome, with some *caveats*. First, cortisol levels will inevitably be increased in critically ill patients, part of the physiological response to stress [58, 59]: On the other hand, a marked increase in serum cortisol –*per se* not used for the diagnosis of Cushing’s syndrome- may be highly suggestive for endogenous hypercortisolism. Indeed, in most patients with infection reported in the literature [48, 60], cortisol levels were 3-to-4 fold higher than normal.

A second argument for caution is the concomitant use of corticosteroids for treatment of the infection. Urinary free cortisol may appear falsely elevated due to cross-reactivity with synthetic steroids, an issue which can be resolved using mass spectrometry [61] rather than immunometric assays on non-extracted urine [62]. 

Lastly, several antibiotics are CYP3A inducers, thus may accelerate hepatic dexamethasone clearance and lead to false positive responses to the overnight suppression test [63]. Should this appear to be the case, measurement of serum dexamethasone may prove useful [64].

The etiological diagnosis is sometimes redundant, as the primary lesion may be apparent at presentation in severe Cushing’s syndrome due to adrenal or ectopic malignancies. Indeed, in severely ill patients, once the suspicion of Cushing’s syndrome has been confirmed, treatment of hypercortisolism should be initiated promptly. Clinical judgement should indicate whether to proceed with the differential diagnosis, await recovery from infection or attempt adrenal imaging, should adrenalectomy appear a viable option. Differential diagnosis of ACTH-dependent Cushing’s syndrome is best performed at expert centers [63].

## Hypercortisolism presenting with thrombotic events

### Pathophysiology

The first report on prothrombotic alterations in the coagulatory cascade in Cushing’s syndrome dates back to the 1980 [65, 66]. Since then, a number of studies reported on hypercoagulability in endogenous hypercortisolism, the most consistent alterations being an increase in clotting factors, in particular factor VIII and von Willebrand factor (vWF), enhanced thrombin generation and reduced fibrinolysis [67–70]. Compensatory elevation of anticoagulants and fibrinolysis activators, such as protein C and plasminogen activator, [68, 69, 71, 72], has also been reported attesting to the complexity of hemostatic abnormalities in Cushing’s syndrome. Biochemical testing reveals shortened aPTT [67], rapid clot formation [73] and prolonged clot lysis time [74].

In addition to hypercoagulability, patients with Cushing’s syndrome also present other features of Virchow’s triad, namely endothelial dysfunction and hemodynamic changes. Endothelial dysfunction has been documented in carotid [75] and coronary arteries [76], possibly due to features such as obesity, diabetes and hypertension [77] in addition to hypercortisolism* per se*. Reduction in blood flow may occur in bedridden patients with severe muscle atrophy or fractures or, in alternative, after surgery. Indeed, environmental triggers for thrombosis, such as reduced mobility and inflammation, proved decisive for the risk assessment of thromboembolic events in patients with Cushing’s syndrome [78]. As for infections, patients with ectopic Cushing’s syndrome and adrenal cancer present the highest risk of thrombotic events due to hypercoagulability induced by malignancy itself [79]. In these cases, thrombosis can affect multiple venuos systems and is associated with lower survival [80].

### Epidemiology

The risk of thrombotic events in patients with Cushing’s syndrome has been quantified in several large retrospective studies [81–83]. Venous thromboembolism occurred in 4–10% of patients with active Cushing’s syndrome [81, 82], regardless of the underlying etiology (Table 2). The standardized incidence ratio for thromboembolic events in the 3 years prior to diagnosis was estimated at 11.5 for patients with Cushing’s disease [83]. Risk of thrombotic events is obviously higher in patients with malignant ectopic or adrenal cancer, given the known association of thromboembolism and neoplasia [84].

**Table 2:** Prevalence of thrombotic events in patients with active Cushing’s syndrome

Study	Number of patients	Indicator
Papakokkinou 2020 [21]	830 person – year for 3 year prior to diagnosis (all CD)	thromboembolism: SIR^a^ 11.5 (C.I. 4.2–25) myocardial infarction: SIR^a^ 4.4 (C.I. 1.2–11.4)
Suarez 2020 [80]	208 patients (168 CD,14 adrenal, 8 ectopic)	12 thromboembolic events (5.7%)
Stuijver 2011 [81]	473 patients (353 CD, 120 adrenal)	17 thromboembolic events (3.6%)
Koutroumpi 2013 [90]	58 patients (43 CD, 13 adrenal, 2 ectopic)	4 thromboembolic events (6.8%)
Manetti 2010 [91]	40 patients (36 CD, 4 adrenal)	1 thromboembolic event (2.5%)
Dekkers 2013 [12]	343 patients (211 CD, 132 adrenal)	thromboembolism: HR^b^ 8.4 (C.I. 3.0–23) myocardial infarction: HR^b^ 2.2 (C.I. 0.5–8.9)
Toivanen 2021 [11]	60 patients (all ectopic)	5 thromboembolic events (8%)
Broder 2015 [22]	1852 patients (all CD)	203 cardiovascular disease/stroke events (11%)
Mondin 2023 [25]	126 patients (all CD)	23 thromboembolic events (18%) 9 cardiovascular events (7%)

CD: Cushing’s disease ^a^SIR: standardized incidence rate compared to general Swedish population; C.I. 95% confidence intervals; ^b^HR: age-sex adjusted hazard ratio compared to general Danish population cohort

No adrenal malignancy was included in reported studies

Venous thromboembolism may also occur after surgery, with percentages ranging from 3 to 12% in patients with Cushing’s disease [81, 82]. Most events occurred within 2 months from transsphenoidal surgery although the standardized incidence ratio remained high even at long-term remission [83].

Arterial thrombotic events, such as myocardial infarction and stroke, represent the major cause of death in patients with Cushing’s syndrome [23, 85]. Indeed, the hazard ratio for myocardial infarction and stroke is 2-to-3-fold than the general population [15, 83, 86]. As with venous thromboembolism, the risk for these events abates but does not appear to entirely disappear after surgery [87].

No data is available on the prevalence of Cushing’s syndrome among patients with venous or arterial thromboembolism. On the other hand, individuals with features of the metabolic syndrome, such as obesity, diabetes, hypertension, are known to present high risk for myocardial infarction and venous thromboembolism [88, 89]. Endogenous hypercortisolism has been shown to underly a small minority of these patients [90, 91] but concurrent thrombotic events may increase the likelihood of Cushing’s syndrome.

### Clinical course

Only scattered cases of Cushing’s syndrome presenting with venous or arterial thrombosis have been reported. Both central, e.g. cavernous sinus [92], retina [93], intestinal [94] and peripheral venous thromboembolism as well as cardiac and peripheral arterial thrombosis [95, 96] were the cause of admission in these reports.

Thromboembolic complications during diagnostic work-up have been reported in a few patients after inferior petrosal sinus sampling [97, 98]; obviously, performing an endovascolar procedure in patients at risk for thromboembolic events carries a higher degree of complications [99]. There have also been reports of disseminated intravascular coagulopathy (DIC) during the initial evaluation for hypercortisolism, usually in context with sepsis or multiple organ infection [100]. Management of the thromboembolic event is most often carried out prior to surgery; in some cases, adrenal steroid synthesis inhibitors were initiated concurrently with anticoagulants. Favourable evolution was reported in most cases, except for the rare patient with malignant tumor or widespread thrombosis.

As mentioned above, thromboembolic events are by far more frequent after surgery, usually within the first weeks from either adrenal or pituitary intervention [81]. All sites may be affected, in some cases even multiple locations in the same patient [101, 102], and possibly even recurring after initial successful treatment [103]. Guidelines for the prevention of postsurgical thromboembolic events are being discussed [104] as is the optimal treatment approach [63, 105, 106].

### Who should be screened

As with patients presenting with infection, the diagnosis of Cushing’s syndrome requires a high degree of suspicion in patients presenting with thromboembolism. Accurate medical history and thorough clinical evaluation are essential to evalutate known risk factors for thromboembolism, such as malignancies, atherosclerotic disorders, drugs with thrombotic potential. Given the rareness of Cushing’s syndrome and the higher prevalence of thrombotic events after surgery, evaluation for endogenous hypercortisolism is not mandated in patients presenting with thrombotic events alone. Clinical suspicion should rest on the presence of other, more specific, features of Cushing’s syndrome [107].

### Diagnostic work-up

Appropriate clinical judgment has to be used during the interpretation of tests for the diagnosis of Cushing’s syndrome in patients presenting acute events such as deep vein thrombosis, pulmonary embolism, cerebral sinus thrombosis or mesenteric ischemia, all conditions reported in patients with pituitary or adrenal tumors. Mild elevations in midnight cortisol or urinary free cortisol may reflect the stress response rather than true endogenous hypercortisolism; the overnight suppression test, while less impaired by stress responses [62], may yield false positives in patients administered drugs accelerating dexamethasone metabolism [63]. In this context, estroprogestins –known prothrombotic drugs- are known to increase serum corticosteroid-binding globulin thus serum cortisol levels, and may impair the interpretation of the overnight suppression test [63]. On note, the suboptimal specificity of screening tests in patients presenting with features of the metabolic syndrome [108, 109], e.g., obesity, diabetes, hypertension –all known risk factors for thrombotic events-, mandates particular caution in the interpretation of test results.

Last, a word of caution on inferior petrosal sinus sampling (IPSS). As mentioned above, an endovascular procedure is inevitably associated with greater risk of complications in patients with endogenous hypercortisolism, Further, the current shortage of corticotropin-releasing hormone has led several centers to administer desmopressin as a stimulatory agent [110]. Desmopressin is used as an hemostatic agent in bleeding disorders and has been shown to stimulate the release of vWF and other endothelial markers in patients with Cushing’s disease [111]. Thus, the risks of performing IPSS with desmopressin should be weighed against the risk of thrombotic events [112], especially in patients with high thrombotic risk profile.

## Conclusions

Both infections and thrombosis are well known complications in Cushing’s syndrome. However, the prevalence of hypercortisolism in these two conditions has never been assessed, a likely consequence of the rarity of endogenous cortisol excess. Uncommon infections without an underlying immunosuppressive condition or apparent idiopathic thrombosis should raise the flag for the presence of cortisol excess and lead to targeted clinical evalutation.

## Electronic supplementary material

Below is the link to the electronic supplementary material.

Supplementary Material 1
