# Correction: Unusual infections and thrombotic events in Cushing’s syndrome

**DOI:** 10.1007/s40618-024-02486-0

**Published:** 2024-11-12

**Authors:** Mattia Barbot, Martina Lazzara, Pierluigi Mazzeo, Francesca Pecori Giraldi

**Affiliations:** 1 Endocrinology Unit, Department of Medicine-DIMED, University Hospital of Padova, Padova, Italy; 2 Department of Clinical Sciences & Community Health, University of Milan, Via Commenda 19, Milano, Italy

**Correction: Journal of Endocrinological Investigation** 10.1007/s40618-024-02454-8

In the original version of this article, some part of the Funding information was missing. The funding information was previously read “Open access funding provided by Università degli Studi di Milano within the CRUI-CARE Agreement.” Should have been “Open access funding provided by Università degli Studi di Milano within the CRUI-CARE Agreement. Open Access funding for this article was provided by Recordati Rare Diseases Italy S.r.l. The funder had no role in the preparation, review, or approval of the manuscript”.

The original article has been corrected.

