# Supplementary material for: Unusual infections and thrombotic events in Cushing’s syndrome
Source: J Endocrinol Invest. 2024 Oct 1;48(Suppl 1):35–43. doi: 10.1007/s40618-024-02454-8 (PMC12031848; doi:10.1007/s40618-024-02454-8)
Supplement: Supplementary file 1 — Supplementary Material 1 [file 40618_2024_2454_MOESM1_ESM.doc]

**Supplemental Table 1** Reported infections in patients with Cushing’s syndrome.

|  | **Main transmission route** | **Type of Infections in CS patients** | **Etiology of CS** |
| --- | --- | --- | --- |
| **Aspergillus fumigatus** | Inhalation of conidia | Pulmonary disease  Aspergilloma  CNS aspergillosis  Disseminated disease  Endophthalmitis [27]  Sinus infection | EAS [26–31]  CD [32–34]  ACC [35] |
| **Cryptococcus neoformans** | Inhalation of spores | Pulmonary  Disseminated disease  Cryptococcal meningitis | EAS [36]  CD [37, 38, 57]  ACC [39, 40] |
| **Nocardias asteroides** | Inhalation of bacteria | Pulmonary disease  Disseminated disease [42] | EAS [26, 41]  CD [43–45] |
| **Pneumocystis jiroveci** | Inhalation of fungus;  Reactivation | Pulmonary disease  ARDS | EAS [52, 61–63]  PBMAH [64]  CD ([65] |

*Abbreviations:; CNS, central nervous system, EAS, Ectopic ACTH secretion; CD, Cushing’s disease; ACC, Adrenocortical carcinoma; ARDS, Acute respiratory distress syndrome; PBMAH, Primary bilateral macronodular adrenal hyperplasia*
